# A deep learning approach in diagnosing fungal keratitis based on corneal photographs

**DOI:** 10.1038/s41598-020-71425-9

**Published:** 2020-09-02

**Authors:** Ming-Tse Kuo, Benny Wei-Yun Hsu, Yu-Kai Yin, Po-Chiung Fang, Hung-Yin Lai, Alexander Chen, Meng-Shan Yu, Vincent S. Tseng

**Affiliations:** 1 Department of Ophthalmology, Kaohsiung Chang Gung Memorial Hospital and Chang Gung University College of Medicine, No.123, Dapi Rd., Niaosong Dist., Kaohsiung, 833 Taiwan, ROC; 2 Department of Computer Science, National Chiao Tung University, No. 1001, Daxue Rd., East Dist., Hsinchu, 300 Taiwan, ROC

**Keywords:** Diagnosis, Physical examination

## Abstract

Fungal keratitis (FK) is the most devastating and vision-threatening microbial keratitis, but clinical diagnosis a great challenge. This study aimed to develop and verify a deep learning (DL)-based corneal photograph model for diagnosing FK. Corneal photos of laboratory-confirmed microbial keratitis were consecutively collected from a single referral center. A DL framework with DenseNet architecture was used to automatically recognize FK from the photo. The diagnoses of FK via corneal photograph for comparing DL-based models were made in the Expert and NCS-Oph group through a majority decision of three non-corneal specialty ophthalmologist and three corneal specialists, respectively. The average percentage of sensitivity, specificity, positive predictive value, and negative predictive value was approximately 71, 68, 60, and 78. The sensitivity was higher than that of the NCS-Oph (52%, *P* < .01), whereas the specificity was lower than that of the NCS-Oph (83%, *P* < .01). The average accuracy of around 70% was comparable with that of the NCS-Oph. Therefore, the sensitive DL-based diagnostic model is a promising tool for improving first-line medical care at rural area in early identification of FK.

## Introduction

Microbial keratitis (MK) is one of the leading causes of blindness^1^. Among MK, fungal keratitis (FK) is the most devasting disease that causes severe vision losses^2, 3^ and accounts for more than half of the MK in several tropical and subtropical regions^4^. Delayed diagnosis may cause deep fungal invasion of cornea and lead to poor penetration of the antifungal agents. The unsuccessful medical treatment of FK may lead to series of severe complications, including corneal melting, glaucoma, and endophthalmitis^5^. Consequently, at least one third of FK patients ultimately underwent surgical interventions such as intrastromal injection of antifungal agents, therapeutic keratoplasty, penetrating keratoplasty, or deep anterior lamellar keratoplasty^6,7^. Therefore, Early diagnosis is essential for avoiding severe complications and minimizing surgical necessities.

Early diagnosis of FK, however, is challenging for first-line physicians or eye care practitioners^8^. The unfamiliarity with the clinical presentation of FK and the lack of diagnostic tests such as culture or polymerase chain reaction often leads to delayed diagnosis and referral to medical center. According to Dahlgren et al.^9^, among the major categories of MK, FK was the most difficult to diagnose by clinical presentations. The sensitivity and specificity of clinical diagnosis of FK were 38% and 45%, respectively. Even corneal specialists were only able to distinguish fungal from bacterial etiology 66% of the time via photographic diagnosis^10^. However, Thomas et al. showed that diagnostic accuracy could be improved by delicate morphological analysis on corneal infiltrates^11^. Therefore, reinforcing clinical image diagnosis in first-line medical services may reduce delayed diagnosis of FK and prevent the development of intractable FK.

Artificial intelligence (AI) based on deep learning (DL) has sparked incredible medical interest globally in recent years. DL is a class of state-of-the-art machine learning techniques, which has no need for manual feature engineering and automatically recognizes the complex structures in high-dimensional data through projection on a low-dimensional manifold^12^. In recent years, DL has been applied to ocular imaging for a variety of purposes such as diagnosing diabetic retinopathy and evaluating optic disc morphologies^13–16^. In view of outstanding performances of DL in ocular imaging, we expect that the application of DL can improve the diagnostic accuracy of FK in primary care and community settings. In consequence, the purpose of this study was to develop a DL-based corneal photo diagnostic model, evaluate the validity of the model, and compare the diagnostic performance of the model with ophthalmologists.

## Materials and methods

### Study design & subjects

This was a retrospective study enrolling patients with clinically suspected MK who had records of corneal digital photographs and laboratory confirmation of microbial invasion. The study adhered to the Declaration of Helsinki and the ARVO statement on human subjects and was approved by the Chang Gung Medical Foundation Institutional Review Board (Ethical approval code: 201800949B0C501). The consent for patients in this study has been waived by the Institutional Review Board. The consecutive patient records in the Kaohsiung Chang Gung Memorial Hospital from June 1, 2007 to May 31, 2018 were retrospectively reviewed. Corneal photography was performed according to the same standard procedure by certified ophthalmic technicians using Nikon D100 camera mounted on Topcon SL-D8 slit lamp biomicroscopy (before May, 2015) and Canon EOS 7D camera mounted on Haag-Streit BX900 slit lamp microscopy (since May, 2015).

The enrolled patients must have received corneal scarping or biopsy and have at least one of the following laboratory confirmations, including direct microscopy (Gram or acid fast stain), culture (blood agar, chocolate agar, Sabouraud dextrose agar, or Löwenstein–Jensen slant), molecular tests (polymerase chain reaction or dot hybridization assay), and pathological examination^17–20^. However, the subjects with mixed infection were excluded. Moreover, the proved subjects without photographic documentation for their MK were excluded. Furthermore, subjects were also excluded if the initial documented photos were obtained after the acute stage of MK (corneal haze without infiltrate and symptoms onset > 7 days for non-mycobacterium bacterial keratitis, > 14 days for FK and herpes keratitis, and > 21 days for mycobacterial keratitis and parasitic keratitis—*Acanthamoeba* keratitis and microsporidial keratitis). One corneal photograph with white light illumination (no slit beam enhancement) for each patient was used for the following experiments. A total of 288 photos were collected from 288 eyes of 288 laboratory-confirmed MK patients.

### Image preprocessing of subjects’ corneal photos

**Figure 1:**
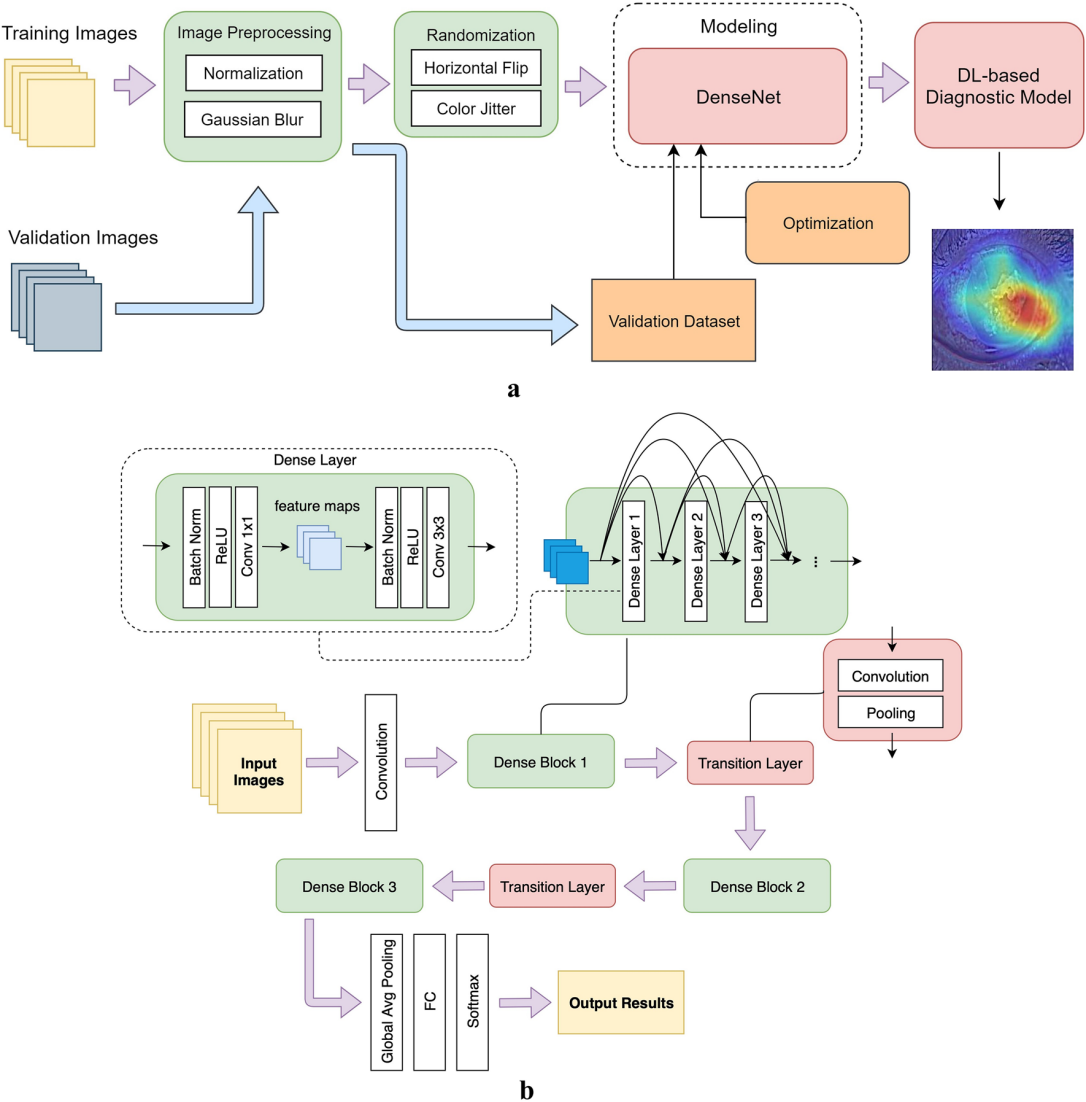
The deep learning framework for diagnosing fungal keratitis by corneal photographs. **(a)** The abstract structure for developing the deep learning-based model for diagnosing fungal keratitis. **(b)** The DenseNet architecture, a deep learning neural network adopted in this study. Images were fed into the first convolution layer and then the output feature was mapped to input the dense block. Dense blocks contain dense networks that connect each layer to every other layer in a feedforward manner. The output of first two dense blocks were the input of transition layers that reduce the dimensions of the channels to prevent further dense blocks from generating too many feature maps. The last dense block produced feature maps, and these maps were fed in the global average pooling layer, fully connected layer, and Softmax to obtain the final classification results. DL, deep learning; ReLU, rectified linear unit; FC, fully connected layer.

All photos used for the development of DL-based model for diagnosing FK were delinked from personal identification except for their corresponding diagnosis. The identification information and date of photography footnoted in the image of each subject were automatically pre-cut with a batch processing manner by a specially designed software. After that, in order to automatically disregarding unnecessary information and reducing the impact of noise from the raw images on DL algorithm, the images were processed with normalization and transformation techniques (Fig. 1). For data normalization, RGB values of each image were calculated for obtaining standard deviation and mean value to normalize each pixel in a range 0 to 1. Gaussian blur, a denoising technique for images, was used to reduce image details to a certain level and make machines unaffected by much noise. After the above image preprocessing, two randomization techniques, horizontal flip and color jitter, were used before entering the training process of the neural network. The processes of horizontal flip and color jitter were built-in functions in the training flow. We set up a random ratio of 0.5, which meant that there was a 50% probability of doing horizontal flip and color jitter on some corneal photos in each training epoch. The randomization techniques were utilized in training process to help the model learn more variations of images.

### Establishment of the DL-based diagnostic model of FK

Convolutional Neural Network (CNN) has been demonstrated to be effective in implementing DL for classifying the image data^21^. Therefore, we exploited the DenseNet algorithm^22^, a representative CNN-based DL method with less computations and more effectiveness than the ResNet algorithm^23^, for establishing DL models for diagnosing FK, of which the framework is shown in Fig. 1. The training dataset was used to teach a DL model how to recognize FK and non-FK photos, while the validation dataset was used to understand the performance of a trained model (Fig. 1a). After the randomization, the model was trained with the DenseNet architecture (Fig. 1b). In order to generate the optimal model, we empirically tuned the hyperparameters of DenseNet, including learning rate, the number of dense blocks, growth rate, and batch size according to the validation results. The visualization technique, Grad-CAM ++ ^24^, was used to realize what regions of the photo were recognized by the DL model.

### Clinical image diagnosis from ophthalmologists of corneal and non-corneal specialty

The clinical diagnostics of FK is based on the clinical features of corneal infiltrates, including feathery or serrated margin, raised slough, colorization, dry texture, and satellite lesion^10,11,25^. Three experienced corneal specialists, who had more than seven years of qualification in the specialty (26 years, 15 years, and 8 years), were asked to provide their clinical impressions for the same corneal photos tested for the DL model for diagnosing FK. Another three senior ophthalmologists of non-corneal specialty with comparable qualifications in clinical practice (25 years, 16 years, and 12 years) were also invited to make their clinical impressions for these photos. The average work experience after ophthalmic qualifications was 16.3 years and 17.7 years for the corneal and non-corneal specialty ophthalmologists (P = 0.8474), respectively. A technician played these digital photos on a 28-inch liquid crystal display monitor to assist all doctors on making their clinical diagnosis in a masked manner. Each doctor was asked to provide one of the two following impressions: presumed FK or presumed non-fungal MK. Expert diagnosis was reached when at least two corneal specialists had the same impression. Similarly, the non-corneal specialty ophthalmologist (NCS-Oph) diagnosis was determined in the same manner.

### Diagnostic validation

**Figure 2:**
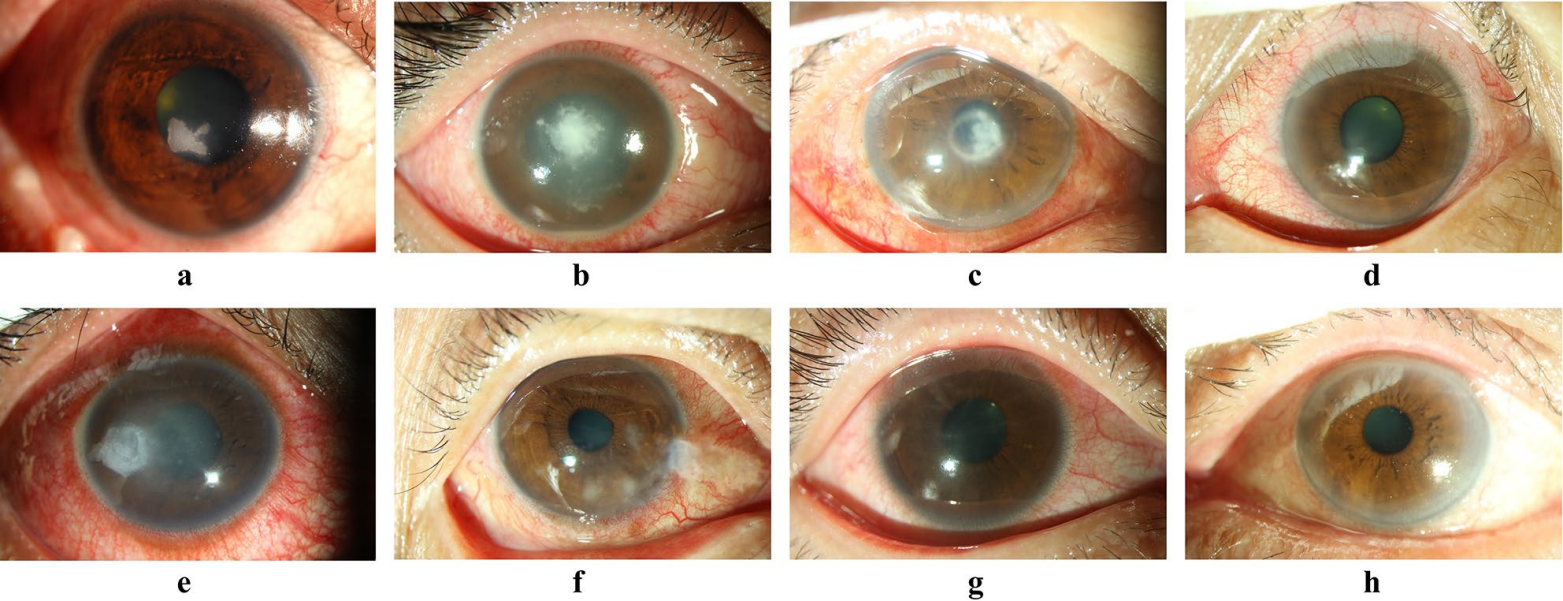
Representative photographs of microbial keratitis caused by fungal and non-fungal pathogens. Fungal keratitis **(a–d)** and non-fungal keratitis **(e–h)**: **(a)** *Candida* keratitis, **(b)** *Fusarium* keratitis, **(c)** *Acremonium* keratitis, **(d)** *Curvularia* keratitis, **(e)** *Pseudomonas* keratitis, **(f)** Herpes keratitis, **(g)** *Acanthamoeba* keratitis, **(h)** Microsporidia keratitis.

Five-fold cross validation was used to assess the AI-assisted diagnostic method for FK. In brief, the photos of MK (Fig. 2) were classified as FK group (n = 114) and non-FK group (n = 174), which included bacterial keratitis (n = 141), herpes keratitis (n = 21), and parasitic keratitis (n = 12). The photos of each group were randomly and equally assigned into five datasets. In turn, four of the datasets were used to fit and train a DL diagnostic model, and the remaining one was used to validate and obtain the scores of performance indices of the model. Finally, five diagnostic models were established and their scores of performance indices were also obtained, respectively. The five models were under the same architecture with different parameters learned by DL models themselves. This fivefold cross validation method was helpful in validating performances on small datasets. All photos were also used to validate the diagnosis of each ophthalmologist, the Expert, and NCS-Oph diagnosis. The average performances of the five DL models, 3 corneal specialty ophthalmologists, and 3 non-corneal specialty ophthalmologists were determined, respectively. The overall performances of the Expert diagnosis and the NCS-Oph diagnosis were estimated, too.

### Statistical analysis

The performance indices, including sensitivity, specificity, positive predictive value (PPV), negative predictive value (NPV), and diagnostic accuracy of FK, were calculated for the DL model, the Expert diagnosis, and the NSC-Oph diagnosis, respectively. The 90% and 95% Wilson/Brown binomial confidence intervals for these indices were estimated. Moreover, the Fisher exact test was performed to identify the statistical difference of each performance index between the two groups. Significant difference was set at *P* < 0.05 and analyzed by GraphPad Prism version 8.2.1 for Windows (GraphPad software, San Diego, CA).

## Results

### Performance of the DL model for diagnosing FK

**Figure 3:**
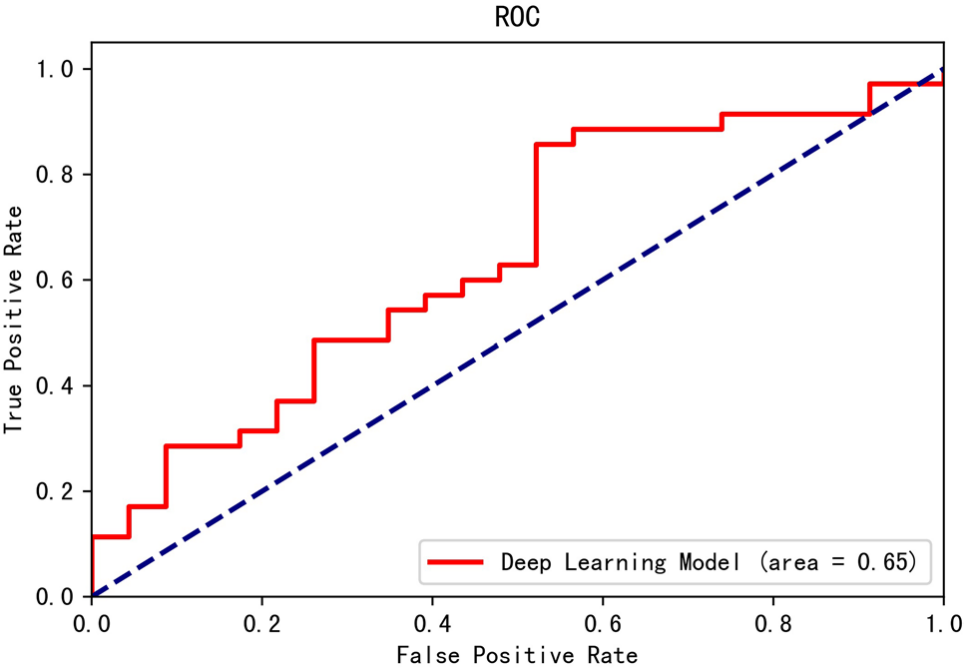
The performance of the deep learning model in differentiating fungal keratitis and non-fungal keratitis was illustrated by the receiver operator characteristic curve. *ROC* receiver operator characteristic curve plot.

The average performance the five DL models for cross validation was shown in Table 1. The average sensitivity and NPV were higher than 70%, while the average specificity and PPV were lower than 70%. The average diagnostic accuracy of the DL model for diagnosing FK was near 70%. The receiver operator characteristic (ROC) curve and area under ROC curve (AUC) were depicted in Fig. 3. The AUC was near 0.65. By using Grad-CAM ++ , we can tell whether the deep learning model learned the correct region of interest or not. We found that the model focused on the cornea of most correct-classified images. However, the discrimination between FK and non-FK on Grad-CAM ++ imaging was not obvious. The crucial point for discriminating FK and non-FK were the complicated combinations of non-linear equations in the model.

**Table 1:** Average performance of the deep learning model in diagnosing fungal keratitis.

Diagnostic performance (95% confidence interval)				
% Sensitivity	% Specificity	% Positive Predictive value	% Negative Predictive value	% Accuracy
71.1	68.4	59.6	78.3	69.4
(62.1–78.6)	(61.1–74.9)	(51.2–67.4)	(71.1–84.1)	(63.9–74.5)

### Performance of the senior ophthalmologists for identifying FK

The average diagnostic performance of the 3 non-corneal specialty ophthalmologists and that of 3 corneal specialty ophthalmologists were shown in Table 2. The average diagnostic sensitivity and NPV of the non-corneal specialty ophthalmologists were significantly lower than those of the corneal specialty ophthalmologists (*P* = 0.0026 and 0.0354, respectively), while the average diagnostic specificity and PPV did not reach statistical difference. The average diagnostic accuracy of the former was significantly lower than that of the latter (*P* = 0.0208).

**Table 2:** Average performance of non-corneal and corneal specialty ophthalmologists in diagnosing fungal keratitis.

Results of ophthalmologists	Diagnostic performance (95% confidence interval)				
	% Sensitivity	% Specificity	% Positive Predictive value	% Negative Predictive value	% Accuracy
Non-corneal specialty	51.8	77.2	59.8	71.0	67.1
	(42.7–60.7)	(70.9–83.3)	(43.1–61.1)	(64.0–76.9)	(62.5–72.3)
Corneal specialty	71.9	78.5	68.7	81.0	75.9
	(63.1–79.4)	(72.0–84.1)	(60.3–76.7)	(74.3–86.1)	(70.7–80.5)

### Performance of the NCS-Oph and Expert diagnosis for identifying FK

Among the 114 patients of FK, the NCS-Oph and Expert diagnosis correctly identified 59 and 81 patients, respectively. Correspondingly, 144 and 143 patients were correctly diagnosed from 174 patients of non-FK. Therefore, the overall sensitivity, specificity, PPV and NPV of the NCS-Oph diagnosis were 52%, 83%, 66%, and 72%, while those of the Expert diagnosis were 71%, 82%, 72%, and 81%, respectively. The overall accuracies of NCS-Oph and Expert diagnosis for FK were 70% and 78%, respectively. For diagnosing FK, the Expert diagnosis was significantly better than NCS-Oph diagnosis in sensitivity (*P* < 0.01), and marginally better in NPV (*P* = 0.051) and accuracy (*P* = 0.057).

### Comparison of the DL model and the ophthalmologist diagnosis in identifying FK

**Figure 4:**
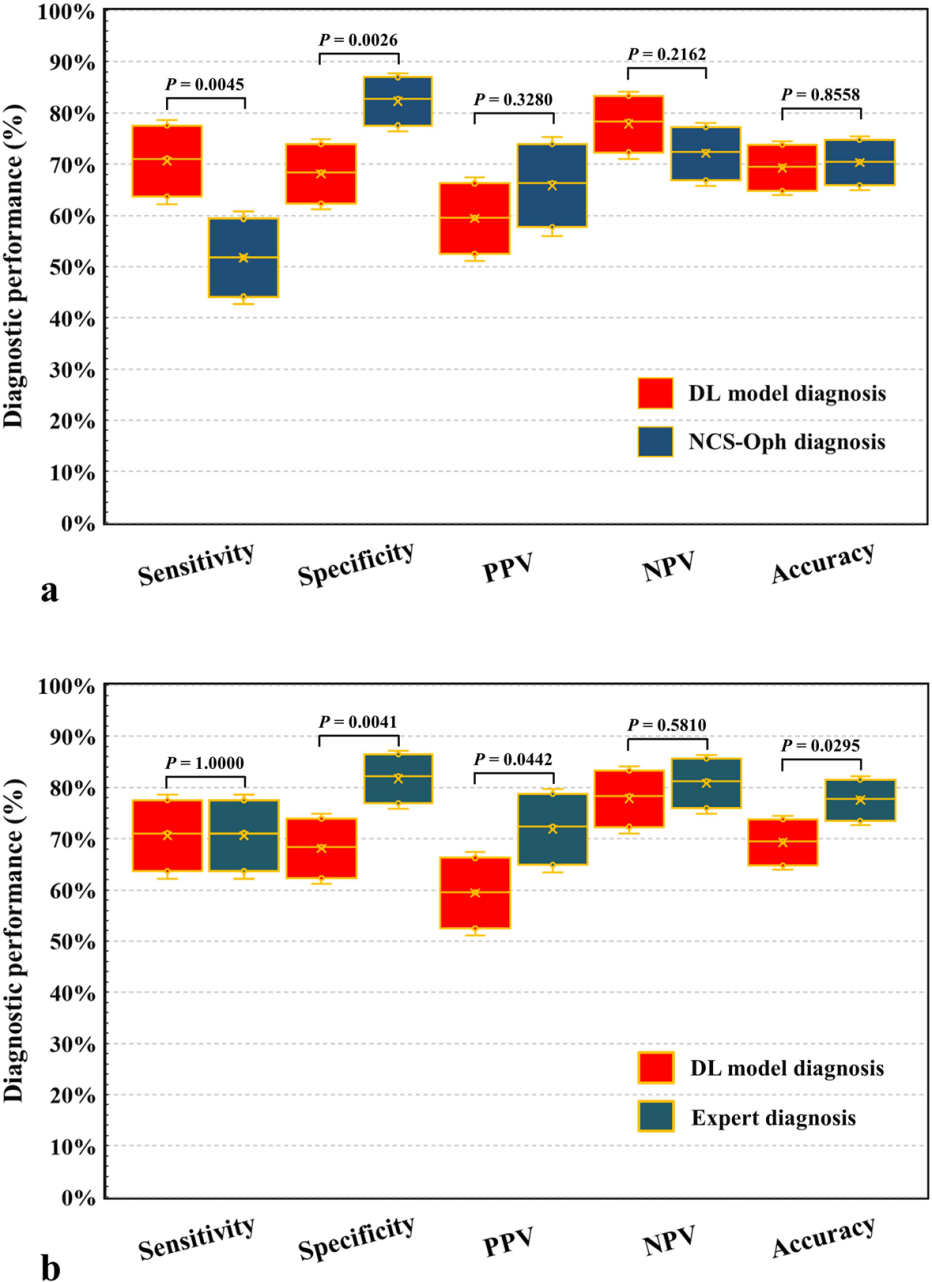
Diagnostic performance of the deep learning model and senior ophthalmologists in the identification of cases of fungal keratitis from total 288 photographs of microbial keratitis. **(a)** Comparing the DL-based model with the NCS-Oph diagnosis. **(b)** Comparing the DL-based model with the Expert diagnosis. *DL* deep learning, *NCS-Oph* non-corneal specialty ophthalmologists, *PPV* positive predictive value, *NPV* negative predictive value; each box was constructed by five parameters, including the mean (center of box), lower and upper 90% confidence limits (floor and top of box), and lower and upper 95% confidence limits (lower and upper error bars). *P* < 0.05 was recognized as statistical difference and determined by two-tailed Fisher exact test.

We compared the average performance of 3 non-corneal specialty ophthalmologists and that of 3 corneal specialty ophthalmologists with the DL model (Table 2). We found the DL models had higher average accuracy than that of the non-corneal specialty ophthalmologists (*P* = 0.8558) but lower than that of the corneal specialty ophthalmologists (*P* = 0.0919). There was no significant difference in the two comparisons. In the average sensitivity, the DL models were significantly higher than that non-corneal specialty ophthalmologists (*P* = 0.0042) and very close to that of the corneal specialty ophthalmologists. However, the DL models were lower than either type of ophthalmologists in the average specificity, especially when compared to corneal specialty ophthalmologists (*P* = 0.0385). We further compared the majority decision of ophthalmologists with the DL model. In comparison to NCS-Oph diagnosis, the DL model had significantly higher sensitivity and lower specificity, but had no significant differences in PPV and NPV (Fig. 4a). However, when compared with the Expert diagnosis, the DL model had significantly lower specificity and PPV, but had comparable performance in sensitivity and NPV (Fig. 4b). Namely, the diagnostic accuracy of DL model is equivalent to that of the NCS-Oph diagnosis, but is significantly lower than that of the Expert diagnosis.

## Discussion

For MK, clinical diagnosis is the most important step to initiate confirmative assays and to provide effective empirical treatment for patients before pathogen confirmation. Clinical diagnosis of FK has been recognized as the most challenging work among MK^9^. The sensitivity, PPV, and diagnostic accuracy based on clinical image were approximately 38%, 45% and 66%, respectively^9,10^. In this study, a novel DL-based diagnostic model for identifying FK was developed based on the corneal photo with direct white light illumination. The average sensitivity, PPV, and diagnostic accuracy of this model were about 71%, 60%, and 70%, respectively (Table 1).

This is the first study to diagnose FK with corneal photographs using DL-based machine learning techniques. Saini et al. used the basic neural network structure to classify infective keratitis^26^. Their input consists of 40 variables on patients’ history and lab data for training and testing. Therefore, the performance of their classification model was based on the completeness of the input variables. In practice, once their model is used to aid clinicians for diagnosis, the clinicians have to wait for the results of lab tests and collect necessary input variables. In contrast, our approach needs only the corneal photos for keratitis classification, such that it offers higher feasibility in real-world applications. Besides, our approach and their model achieve comparable performance in terms of diagnostic accuracy. However, they used only 63 cases for training and 43 cases for testing. Also, they did not use cross validation to minimize the bias, which may exist in the distribution of training and testing datasets. In contrast, we applied fivefold cross validation to validate the robust performance of our model. By observing the misclassified images, we found that most of the misclassifications were not because the model misclassified the classes of keratitis. Instead, it was due to incorrect focusing of unwanted regions of the photo, such as eyelid, eyelash, and sclera of the eye. The major reason for misclassifications of keratitis lies in the limited number of training data, which made the model incapable of identifying the region of interest. We plan to solve the above problems in the future through approaches like increasing the number of images for training, utilizing more machine learning techniques like transfer learning, and applying region-of-interest detection method. We expect the misclassifications to be reduced significantly through these reinforcements. Although the average diagnostic performance of DL models was not as good as the overall performance of Expert diagnosis, the DL model has comparable diagnostic accuracy with the NCS-Oph diagnosis (Fig. 4). This result implies that DL diagnostic model may be a practical tool in a primary care or emergency services, where patients with MK are often present. In clinical practice, the false-negative diagnosis for FK may lead to delayed diagnosis and consequently, a disastrous visual prognosis that is worse than expected since anti-fungal agents are not usually prescribed as a part of empirical treatment for MK.

Since most eye care practitioners are inexperienced with FK, delayed diagnosis is common and early morphological features are easily missed, resulting in poor clinical diagnosis. Therefore, many on-demand laboratory tests are being developed to increase the diagnostic accuracy of FK^27^. Corneal scrapes for direct microscopy and microbial culture are commonly applied in medical referral centers but often results in low sensitivity due to inadequacy of tissue sampling, examination by novice examiners, and confrontation of fastidious microorganisms^28,29^. Therefore, several highly sensitive DNA-based molecular tests have been developed in the past decade^17,30,31^. However, these molecular tests often need sophisticated instruments or heavy laboratory procedures. In vivo confocal microscopy provides an alternate way for identifying FK^32^, but the diagnostic sensitivity was only moderate (71%) even for experienced observers^33^. An AI approach with an image recognition algorithm combined texture analysis with support vector machine was therefore adopted to improve the performance of confocal microscopy^34^.

Despite drastic improvements in laboratory diagnostic tests for FK, clinical image diagnosis is still irreplaceable. In reality, first impression of FK is almost always made with clinical images and consequently affects the decisions on the ordering of laboratory tests, prescription of medications, and referral to medical center. AI-assisted diagnosis in confocal microscopy shows high potential in diagnosing FK through a machine learning technique^34^; however, this equipment is unpopular even in medical centers due to low cost-effectiveness. Furthermore, ophthalmologists are already scarce in rural areas, not to mention corneal specialists. Therefore, the refinement of DL model for immediate image diagnosis of FK can definitely benefit primary care practitioners and rural patients.

There are some potential limitations in this study. The compliance during photographing may be reduced due to pain and photophobia in patients with MK, probably resulting in poorer image quality for inexperienced photographers. In addition, a corneal photograph is more complex than a fundal photograph due to prominent light reflections (Fig. 2), which might influence the training quality and diagnostic performance of a developed DL model. Nonetheless, poor image quality reflects the real-world challenges and put the robustness of DL diagnostic model into test. By increasing the training datasets, DL-based models can become more robust, but the amount of dataset needed to reach this top level of performance is still inconclusive. For FK, corneal image data is probably limited by high dimension and small sample size. By including massive image data, Andre Esteva et al. trained a DL model to classify skin tumor reaching dermatologist-level classification of skin cancer with an accuracy rate of 91%^35^. Thus, we believe a future study collecting more MK photographs from several medical centers will refine and greatly reinforce the current DL model. Moreover, before the DL model is adopted as a primary care device, a prospective study for diagnosing FK patients will be also required for external validation.

In conclusion, the performance of the DL model for diagnosing FK was better than the previously reported diagnostic performance of ophthalmologists and comparable to that of the NCS-Oph diagnosis. In addition, the DL model had better sensitivity than that of the NCS ophthalmologist. This result suggests the current DL model can help FK in clinical practice, especially in primary care units or rural area. The clinician can use it as an adjunctive test with personal diagnostic experience and historical information of a patient to increase the diagnostic sensitivity of FK. Hence, we anticipate a robust and clinically useful AI device for diagnosing FK by including more training dataset with high image qualities of MK, historical variables, and integrating multiple models.
